# The Extraction of Simple Relationships in Growth Factor-Specific Multiple-Input and Multiple-Output Systems in Cell-Fate Decisions by Backward Elimination PLS Regression

**DOI:** 10.1371/journal.pone.0072780

**Published:** 2013-09-09

**Authors:** Yuki Akimoto, Katsuyuki Yugi, Shinsuke Uda, Takamasa Kudo, Yasunori Komori, Hiroyuki Kubota, Shinya Kuroda

**Affiliations:** 1 Department of Biophysics and Biochemistry, Graduate School of Science, University of Tokyo, Hongo, Bunkyo-ku, Tokyo, Japan; 2 Department of Computational Biology, Graduate School of Frontier Sciences, University of Tokyo, Hongo, Bunkyo-ku, Tokyo, Japan; 3 CREST, Japan Science and Technology Corporation, Bunkyo-ku, Tokyo, Japan; University of Ulm, Germany

## Abstract

Cells use common signaling molecules for the selective control of downstream gene expression and cell-fate decisions. The relationship between signaling molecules and downstream gene expression and cellular phenotypes is a multiple-input and multiple-output (MIMO) system and is difficult to understand due to its complexity. For example, it has been reported that, in PC12 cells, different types of growth factors activate MAP kinases (MAPKs) including ERK, JNK, and p38, and CREB, for selective protein expression of immediate early genes (IEGs) such as c-FOS, c-JUN, EGR1, JUNB, and FOSB, leading to cell differentiation, proliferation and cell death; however, how multiple-inputs such as MAPKs and CREB regulate multiple-outputs such as expression of the IEGs and cellular phenotypes remains unclear. To address this issue, we employed a statistical method called partial least squares (PLS) regression, which involves a reduction of the dimensionality of the inputs and outputs into latent variables and a linear regression between these latent variables. We measured 1,200 data points for MAPKs and CREB as the inputs and 1,900 data points for IEGs and cellular phenotypes as the outputs, and we constructed the PLS model from these data. The PLS model highlighted the complexity of the MIMO system and growth factor-specific input-output relationships of cell-fate decisions in PC12 cells. Furthermore, to reduce the complexity, we applied a backward elimination method to the PLS regression, in which 60 input variables were reduced to 5 variables, including the phosphorylation of ERK at 10 min, CREB at 5 min and 60 min, AKT at 5 min and JNK at 30 min. The simple PLS model with only 5 input variables demonstrated a predictive ability comparable to that of the full PLS model. The 5 input variables effectively extracted the growth factor-specific simple relationships within the MIMO system in cell-fate decisions in PC12 cells.

## Introduction

Cells use common signaling molecules to selectively control downstream gene expression and cell-fate decisions. The relationship between signaling molecules and gene expression or cellular phenotypes was previously thought to be a one-to-one correlation. However, recent studies have revealed that signaling molecules and downstream gene expression levels and cellular phenotypes are mutually connected, and their relationship appears to be a multiple-input and multiple-output (MIMO) system [1]–[6].

For example, PC12 cells, an adrenal chromaffin cell line, have been shown to undergo cell differentiation, proliferation and death in response to various growth factors [7]–[11]. Nerve growth factor (NGF) and pituitary adenylate cyclase-activating polypeptide (PACAP) induce differentiation and neurite extension, epidermal growth factor (EGF) induces cell proliferation, and the protein synthesis inhibitor anisomycin induces cell death [9]–[18]. These stimuli use common signaling pathways. NGF induces differentiation via the receptor-tyrosine kinase, TrkA, which causes a sustained activation of downstream signaling pathways, including both the ERK and AKT pathways [9], [10], [19]. PACAP activates the G protein type receptor PAC1, which phosphorylates CREB through cAMP-dependent protein kinase A (PKA) activation, leading to cell differentiation [10], [20], [21]. EGF induces cell proliferation by activating the tyrosine kinase receptor EGFR, which transiently activates the ERK and AKT pathways [9], [15], [22], [23]. Anisomycin activates mitogen-activated protein kinase (MAPK) cascades, such as JNK and p38, as well as caspases, including Caspase 3, which leads to cell death. Moreover, signaling molecules transmit information downstream via the protein expression of immediate early genes (IEGs), including c-Fos, c-Jun, EGR1, FosB and JunB [24], [25]. Thus, a wide range of stimuli encode information into specific temporal patterns and combinations of the multiple-inputs, such as MAPKs and CREB, that are further decoded by the multiple-outputs, such as expression of IEGs to exert biological functions in PC12 cells. However, the essential and simple relationship in the MIMO system remains to be elucidated.

To analyze the MIMO system between signaling molecules and cellular phenotypes, a statistical analysis called partial least square (PLS) regression has been applied to apoptotic signaling pathways [1]–[3], [26]–[28]. The application of PLS regressions to the MIMO system involve reducing the dimensionality of the inputs and outputs into latent variables, which are selectively weighted linear combinations of the inputs and outputs. A linear regression is then performed between the latent variables of the inputs and the outputs. Because the latent variables explain the characteristics of the data using a smaller number of latent variables than the number of original variables, those latent variables are called principal components. This method can relate multiple signaling molecules to multiple downstream functions based on heterogeneous multivariate signaling in response to various stimuli. The principal components in the PLS model consist of linear combinations of all variables. Because the number of variables is not reduced and complexity still remains, the result of the PLS regression is difficult to intuitively understand. To facilitate a better understanding of the MIMO system, a method for further reducing the number of variables is required.

In this study, we employed PLS regression and analyzed the complex relationship between the phosphorylation of signaling molecules and the expression of IEGs and cellular phenotypes in PC12 cells in response to various stimuli. The PLS model highlighted the complex characteristics of the MIMO system and stimuli specific input-output relationships of cell-fate decisions in PC12 cells. Furthermore, to reduce the number of input variables in the PLS model, we applied a backward elimination method to the PLS regression model and obtained a simple PLS model with 5 input variables. The simple PLS model with only 5 input variables demonstrated a predictive ability comparable to that of the full PLS model with 60 variables. The 5 input variables effectively highlight the simple relationships within the MIMO system and stimuli specific input-output relationships of cell-fate decisions in PC12 cells. the simple relationships can be intuitively understood and easily observed by visual inspection.

## Results

### The Multiple-input and Multiple-output System in PC12 Cells

**Figure 1:**
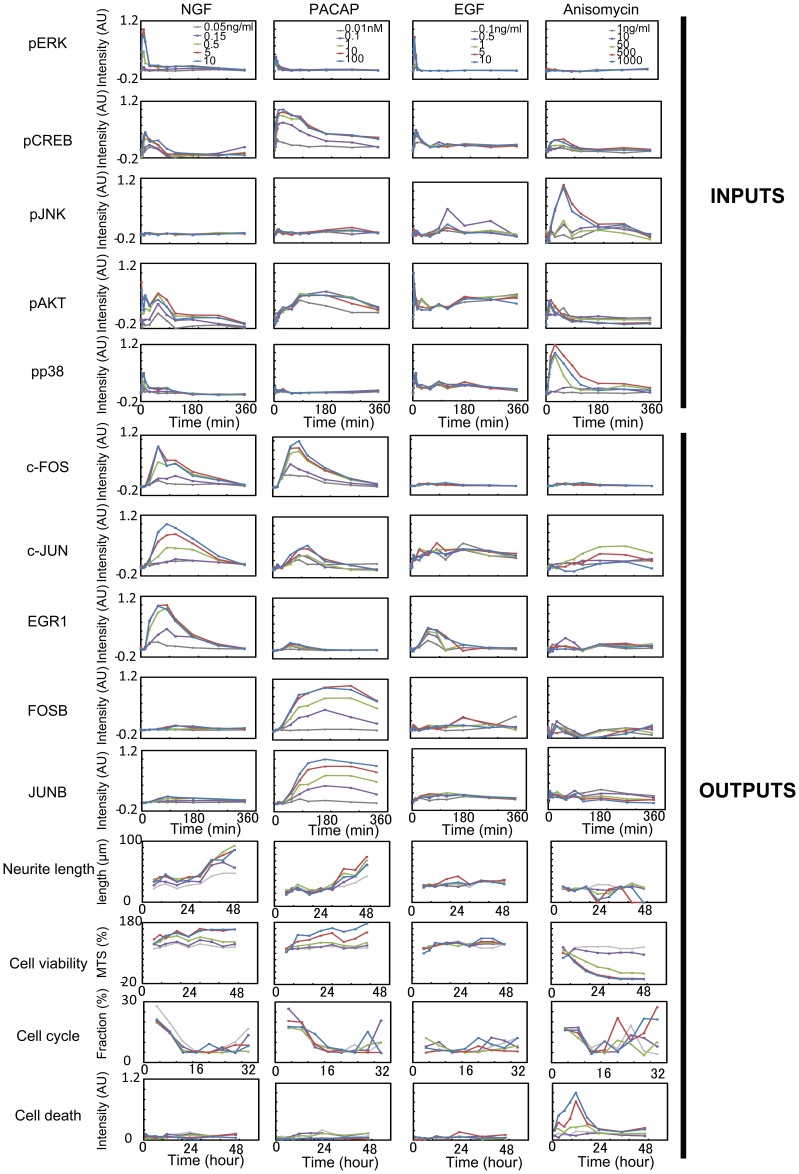
The inputs and outputs in the MIMO system for cell-fate decisions in PC12 cells. The inputs consisted of the 12 time points of pERK, pCREB, pJNK, pAKT, and pp38 in response to 5 doses of 4 stimuli (Tables 1 and S2). The outputs consisted of the 12 time points for protein expression of c-FOS, c-JUN, EGR1, FOSB, and JUNB, and 9 time points for the neurite lengths, cell viability (respiratory chain activity of mitochondria), cell cycle (S-phase fraction) and cell death (Caspase3 activity) in response to 5 doses of 4 stimuli (Tables 1 and S1). The doses of the growth factors are indicated by different colors.

We stimulated PC12 cells with various doses of NGF, PACAP, EGF, and anisomysin and measured time series data of the phosphorylation of signaling molecules, including ERK (pERK), CREB (pCREB), JNK (pJNK), AKT (pAKT) and p38 (pp38) (Figs. 1, Table S1), protein expression levels of immediate early genes (IEGs), including c-FOS, c-JUN, EGR1, JUNB and FOSB (Fig. 1, Table S1), and cellular phenotypes, including neurite lengths, cell viability (respiratory chain activity of mitochondria), cell cycle (S-phase fraction) and cell death (Caspase3 activity) (Figs. 1, Table S1). Among many asssays for detection of apoptosis, we chose caspase3 activity because of the availability for high-throughput assay. NGF, PACAP, EGF, and anisomycin induced distinct temporal patterns and combinations of phosphorylation of signaling molecules, IEGs and cellular phenotypes. We did not observe obvious cell proliferation by EGF stimulation under the conditions. Here, we regarded the phosphorylation of signaling molecules as the inputs and the IEGs and cellular phenotypes as the outputs.

### Construction of the PLS Model

**Figure 2:**
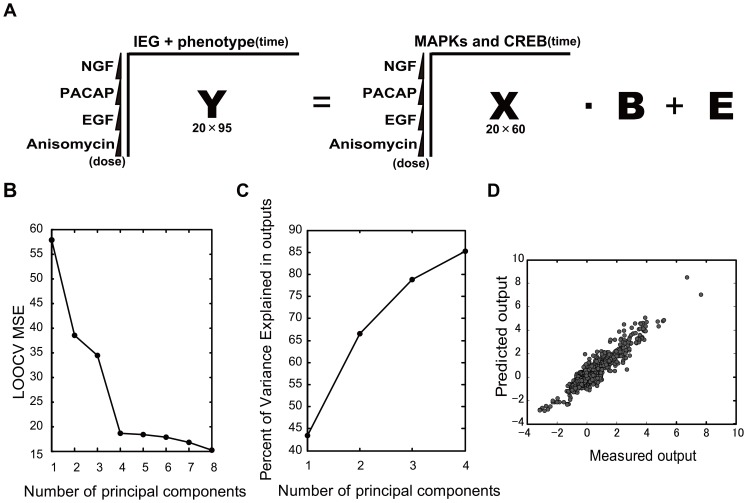
Construction of the PLS model. (**A**) Construction of the PLS model. Inputs matrix **X** (20×60) regressed against the outputs matrix **Y** (20×95). Each column and row in **X** correspond with time course points of MAPKs and CREB, and the doses of stimuli, respectively. Each column and row in **Y** correspond with time course points of the IEGs and phenotypes, and with doses of stimuli, respectively. **B** is the coefficient matrix and **E** is the residue matrix of the PLS model. (**B**) LOOCV MSE (leave-one-out cross validation mean square error) as a function of the number of principal components. (**C**) The cumulative contribution percentage of the principal components. (**D**) Correlation plots between the measured and predicted outputs. The Pearson correlation coefficient, *r*, was 0.94. Each dot represents a single time point for one of the outputs.

We applied PLS regression to infer the MIMO system underlying cell-fate decisions in PC12 cells (Fig. 2) [29]. PLS regression is a regression method for use with MIMO systems that involve reducing the dimensionality of the inputs and outputs into latent variables, which are selectively weighted linear combinations of the inputs and the outputs, denoted as principal components. A linear regression is then performed between the principal components of the inputs and the principal components of the outputs (see Materials and Methods). The principal components were determined to maximize the capture of the covariance between the input latent variable and the output latent variable, and the principal components were orthogonal to one another. Thus, the PLS regression predicts multiple output variables from multiple input variables.

The input data set consisted of 20×60 matrices of phosphorylation of signaling molecules at 12 time points (60 variables) that involved 5 doses of 4 stimuli (Fig. 2A, Tables 1 and S1, see Materials and Methods). The output dataset consisted of 20×95 matrices of the protein expression of 5 IEGs with 12 time points and cellular phenotypes of neurite lengths, cell viability and cell death at 9 time points and cell cycle at 8 time points (95 variables) that involved 5 doses of 4 stimuli (Fig. 2A, Tables 1 and S1, see Materials and Methods). We used the LOOCV MSE (leave-one-out cross validation mean squared error) [30] as the estimated prediction error to optimize the number of model dimensions (Fig. 2B) and determined that the LOOCV MSE was minimized with four principal components. The first principal component captured approximately 45% of the total variance, the first and second principal components captured 67% of the total variance, and the first to fourth principal components captured approximately 85% of the total variance (Fig. 2C). The Pearson correlation coefficient between the measured outputs and the predicted outputs in the four principal components was 0.94 (Fig. 2D).

**Table 1:** Summary of the input (1,200 points) and output (1,900 points) data shown in Fig. 1.

	Molecule/Phenotype	Time points
Input	pERK	0, 2, 5, 10, 15, 30, 60, 90, 120, 180, 270, 360 (min)
	pCREB	
	pAKT	
	pJNK	
	pp38	
Output	c-FOS	0, 2, 5, 10, 15, 30, 60, 90, 120, 180, 270, 360 (min)
	FOSB	
	c-JUN	
	EGR1	
	JUNB	
	Neurite length	6, 9, 12, 18, 24, 30, 36, 42, 48 (hour)
	Cell viability	6, 9, 12, 18, 24, 30, 36, 42, 48 (hour)
	Cell death	1, 3, 6, 9, 12, 18, 24, 36, 48 (hour)
	Cell cycle	4, 8, 12, 16, 20, 24, 28, 32 (hour)

PLS regression characterizes the input-output system using “loadings”, which are the vector projections of the unit direction vector of the principal component on each variable, and “scores”, which are the projections of sample points on the principal component direction. In short, loadings represent the contribution of each variable to the principal component, and scores represent condition specificity in the principal component.

**Figure 3:**
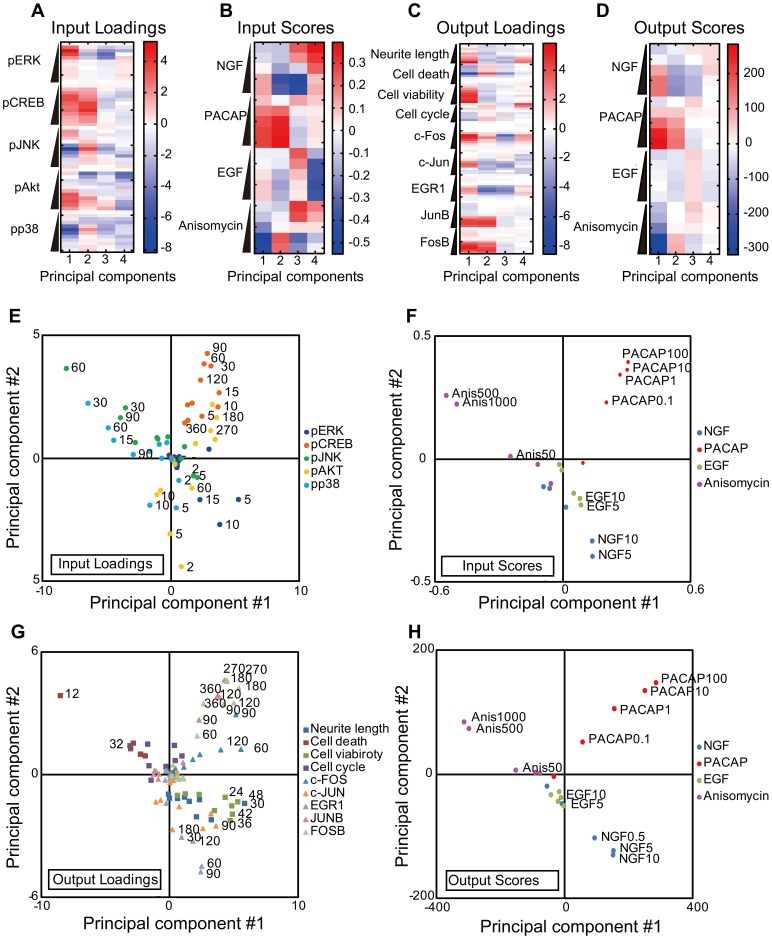
Loadings and scores of the principal components of the inputs and outputs. (**A**) Input loadings. A wedge indicates the temporal evolution of the indicated molecules from 0 to 360 min (Table 1). (**B**) Input scores. A wedge indicates the doses of the stimulant. (**C**) Output loadings. A wedge indicates the temporal evolution (Table 1). (**D**) Output scores. A wedge indicates the doses of the indicated molecules (Fig. 1). The red and blue colors indicate positive and negative values, respectively (**A**–**D**). Scatter plots of input loadings (**E**), input scores (**F**), output loadings (**G**) and output scores (**H**) of the first and second principal components. The colors correspond to the latent variables (**E**, **G**) and stimuli (**F**, **H**). The numbers indicate the time (minute for pERK, pCREB, pJNK, pAKT, pp38, c-FOS, c-JUN, EGR1, JUNB and FOSB and hour for neurite lengths, cell viability, cell cycle and cell death).

In the input loadings of the first principal component, pERK, pCREB and pAKT were positive, whereas pJNK and pp38 were negative (Fig. 3A), indicating their opposing contributions to the first principal component. In the input scores of the first principal component, NGF, PACAP and EGF were positive, whereas anisomycin was negative (Fig. 3B), indicating that anisomycin was inversely correlated to the other growth factors in the first principal component. In the output loadings of the first principal component of the outputs, the neurite length, cell viability, and all IEGs were positive, whereas cell death and cell cycle were negative (Fig. 3C). In the output scores, NGF and PACAP were positive, whereas anisomycin was negative (Fig. 3D), indicating that anisomycin was inversely correlated with the growth factors in the first principal component. These results indicate that the first PLS component divided the data into cell survival/differentiation and cell death. In the input loadings of the second principal component, pCREB and pJNK, late pAKT, and pp38 were positive, whereas pERK and early pAKT were negative. In the input scores, PACAP and anisomycin were positive, whereas NGF and EGF were negative. In the output loadings of the second principal component, cell death, cell cycle and c-FOS, JUNB and FOSB were positive, whereas the neurite length, cell viability, c-JUN and EGR1 were negative. In the output scores, PACAP and anisomycin were positive, whereas NGF and EGF were negative. These observations indicate that the second principal component divided the data into ligands of receptor-type tyrosine kinase (NGF and EGF) and those of other receptor types (PACAP and anisomycin). The third principal component divided the data into higher and lower doses of stimuli, and the fourth principal component divided the data into EGF and others.

The first and second principal components captured approximately 67% of the variance (Fig. 2C), and we plotted both the loadings and scores on these two principal components (Fig. 3E–H). In the first quadrant of the loadings, pCREB and late pAKT in the inputs were correlated with c-FOS, JUNB and FOSB in the outputs (Fig. 3E, G). In the second quadrant of the loadings, pJNK and pp38 in the input were correlated with cell death and cell cycles in the outputs (Fig. 3 E, G). In the fourth quadrant, pERK and early pAKT in the inputs were correlated with the neurite length, cell viability, EGR1 and c-JUN in the outputs. In the scores, the first, second, and forth quadrant involved PACAP, anisomycin and NGF, respectively (Fig. 3F, H), indicating that these quadrants represent stimuli-specific input-output relationships. Thus, the loadings and scores of the first and second principal components highlight characteristics of the MIMO system and growth factor specific input-output relationships of cell-fate decisions in PC12 cells, respectively.

### Validation of the PLS Model

We validated the PLS model using additional experimental data including inhibitors of signaling molecules. We perturbed the activity of signaling molecules by adding inhibitors and measured the inputs and cellular phenotypes (Table S2). We used PD0325901 (MEK inhibitor), H89 (PKA inhibitor), LY294002 (PI3K inhibitor), SB203580 (p38 inhibitor), and SP600125 (JNK inhibitor) that are thought to inhibit pERK, pCREB, pJNK, pp38 and pAKT, respectively.

**Figure 4:**
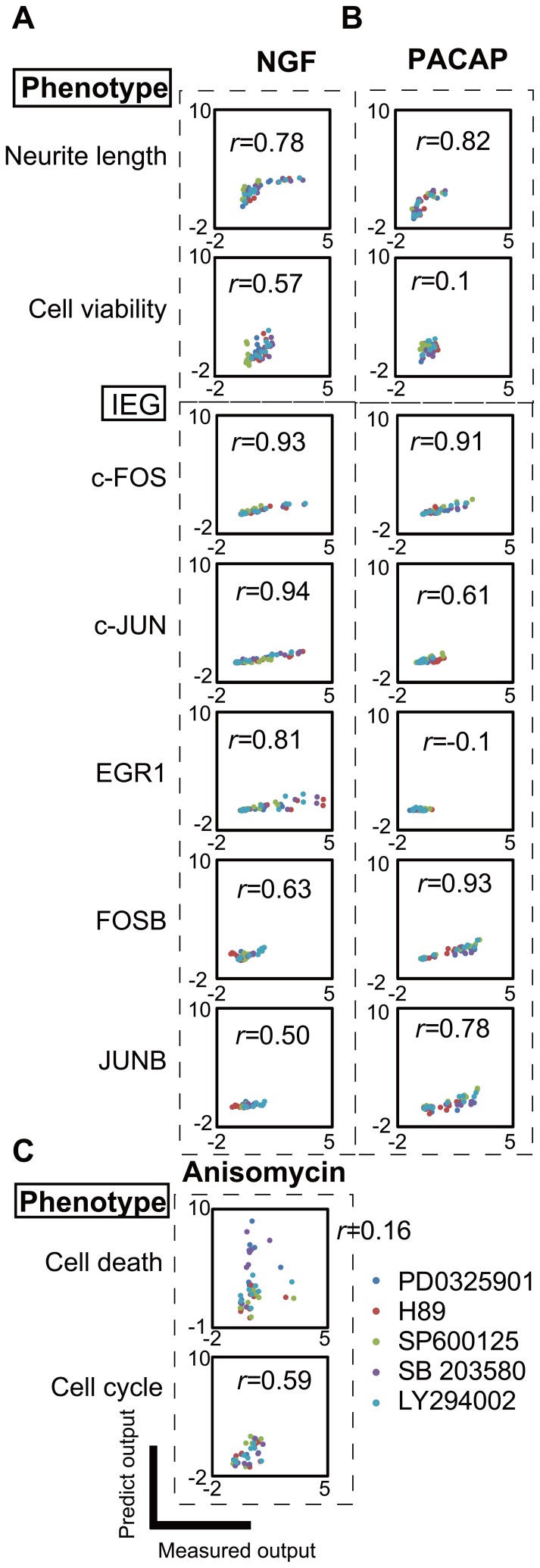
Validation of the PLS model using inhibitor experiments. Correlation plots between the measured outputs and predicted outputs with NGF (**A**), PACAP (**B**) and anisomycin (**C**). The correlation coefficient, *r*, is indicated in each plot. Each dot represents a single time point. The data sets with PD0325901 (MEK inhibitor), H89 (PKA inhibitor), LY294002 (PI3K inhibitor), SB203580 (p38 inhibitor), SP600125 (JNK inhibitor) (Table S2) are indicated by the various colors.

Because of prominent effects of NGF and PACAP on neurite lengths and MTS, and of anisomycin on cell cycle and cell death, we chose these stimuli for validation by the inhibitor experiments. Using the measured inputs in the presence of the inhibitors, the PLS model predicted the neurite length in the presence of the inhibitors (Fig. 4A, B). The predicted neurite length showed a high correlation (*r
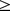
*0.7) with the measured neurite length in response to NGF (*r* = 0.78) and PACAP (*r* = 0.82). The predicted c-FOS, c-JUN and EGR1 expression levels showed high correlations with the measured data in response to NGF (*r* = 0.93 for c-FOS, *r* = 0.94 for c-JUN, *r* = 0.81 for EGR1) (Fig. 4A, B). The predicted c-FOS FOSB and JUNB expression levels were highly correlated with the measured data in response to PACAP (*r* = 0.91 for c-FOS, *r* = 0.93 for FOSB, *r* = 0.78 for JUNB) (Fig. 4B). However, we observed a low correlation (*r
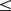
*0.5) between JUNB and NGF (*r* = 0.50) and between EGR1 and PACAP (*r* = 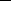
0.1) (Fig. 4A, B). This lack of correlation may be attributable to the low expression levels of the IEGs (Table S2) rather than a low predictive ability of the PLS model. Thus, the PLS model predicted data that correlated well with the inhibitor experimental data regarding the neurite length and protein expression levels of the IEGs. A low correlation between cell viability and PACAP (r = 0.1) may be caused by small changes of cell viability, similarly to the low correlations of some IEGs. A low predictive ability for cell death (r  = 0.16 for anisomycin) may occur due to the complex and drastic response only in the presense of inhibitors, and thus the model could not be trained well in the absence of inhibitors.

We previously found that simultaneous addition of NGF and PACAP leads to synergistic induction of FOSB and JUNB [31]. We validated our model by the experiment with simultaneous addition of NGF and PACAP, and found that the model reasonably predicted the induction of FOSB and JUNB (Table S6, Figure S2). Thus, the PLS model predicted data that correlated well with the additional experimental data. Considering the assumption of the linear relationship in the PLS regression, this suggests that the synergistic effect could lie between receptors and the inputs, rather than between the inputs and outputs of the MIMO system, and that there is no strong nonlinear cross-talk in the MIMO system.

### Reduction of the PLS Model by Backward Elimination PLS Regression

**Figure 5:**
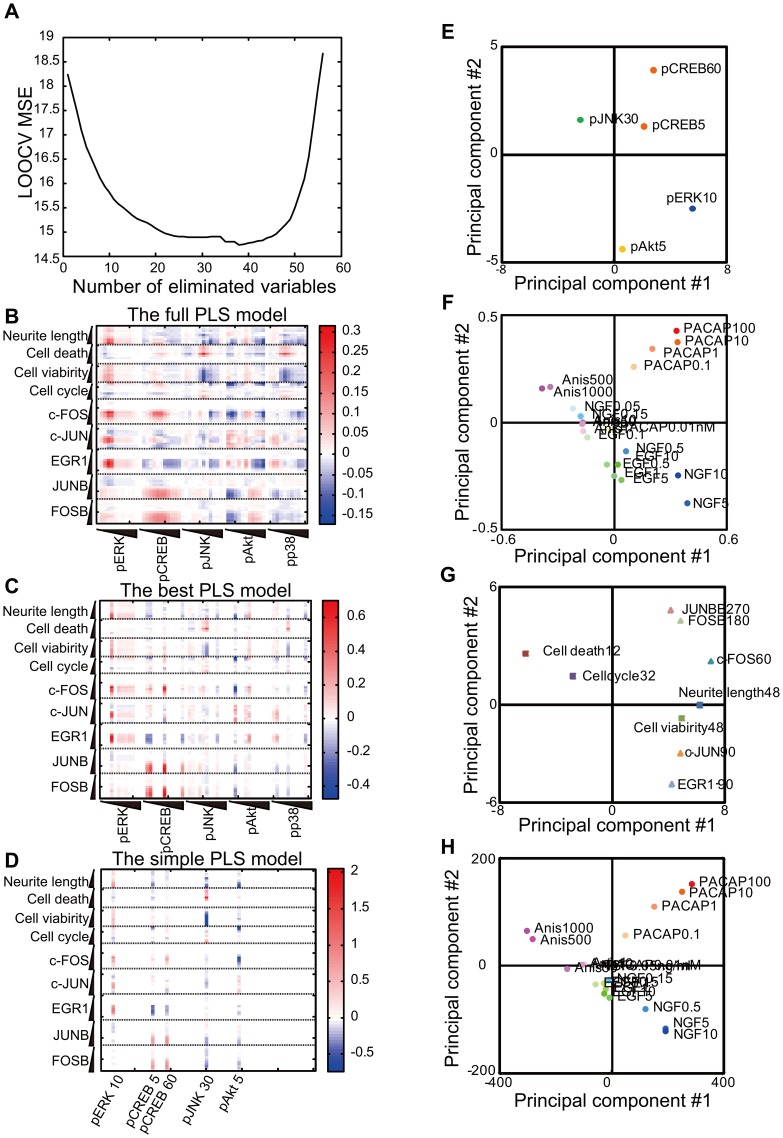
Reduction of the PLS model by backward elimination PLS regression. (**A**) MSE of LOOCV as a function of number of the eliminated variables via the backward elimination PLS regression. Coefficient matrix of the full PLS model with 60 input variables (**B**), the best PLS model with 22 input variables (**C**) and the simple PLS model with 5 input variables (**D**). The red and blue colors indicate positive and negative values, respectively. As the number of the variables reduced, the contribution of remained variables relatively increased, and as a result, magnitude of the regression coefficient increased. The scatter plots of the input loadings (**E**), input scores (**F**), output loadings (**G**) and output scores (**H**) of the first and second principal components of the simple PLS model. The colors correspond to the latent variables (**E**, **G**) and stimuli (**F**, **H**).

**Figure 6:**
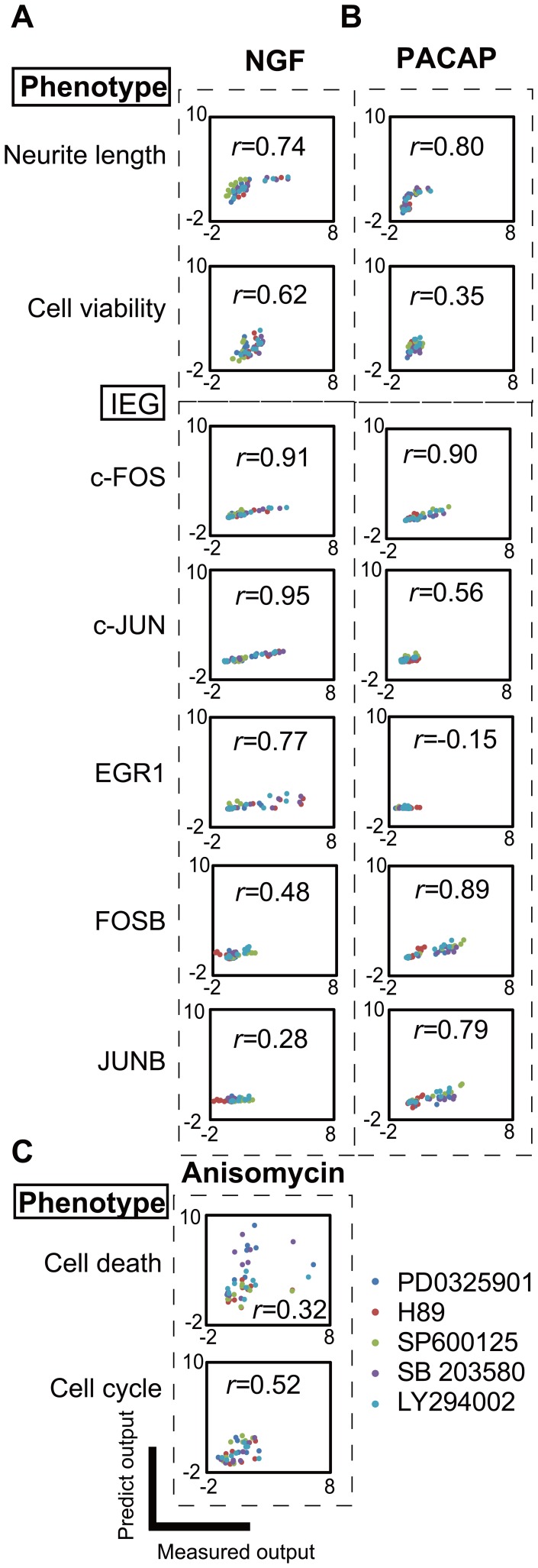
Validation of the simple PLS model using inhibitor experiments. Correlation plots between the measured outputs and predicted outputs using same experiment as Figure 4 with NGF (**A**), PACAP (**B**) and anisomycin (**C**).

**Figure 7:**
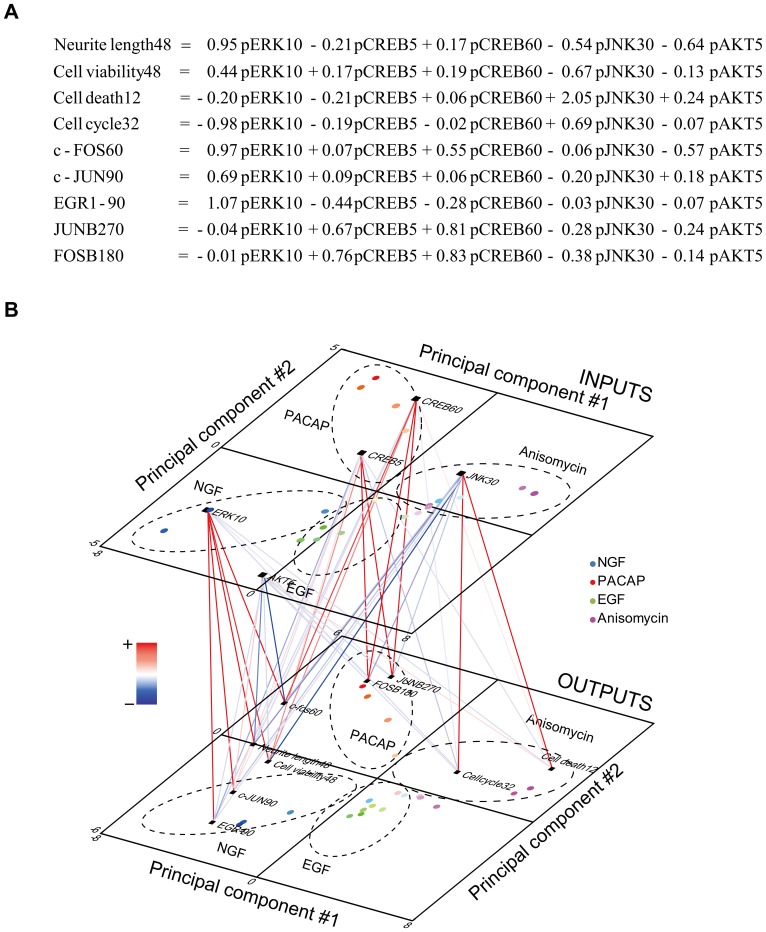
The simple relationships in the MIMO system in cell fate decision in PC12 cells. (A) The simple PLS model with 5 input and 9 output variables. (B) Loadings (squares) and scores (circles) of the first and second principal components in the simple PLS model with 5 input and 9 output variables were bi-plotted in input and output layers. Lines across the layers are coefficients of the matrix of the simple PLS model whose values are indicated by the color bar. The colors of circles indicate the stimuli.

Although PLS regression involves reducing the dimensionality of the inputs into principal components, the principal components continue to involve all of the inputs, making it difficult to intuitively understand how the combination of inputs correlates with the outputs. To facilitate an intuitive understanding of the relationship by reducing the number of variables, we employed a backward elimination variable selection process [32] in the PLS regression called the backward elimination PLS regression method. We constructed a set of single variable-eliminated PLS models and estimated the LOOCV MSE of each model. We eliminated the variable with the minimum LOOCV MSE. We then reconstructed each single variable-eliminated PLS model using the remainder of the variables. We iterated this step sequentially and we maintain the number of components at four through the whole process. As a result, as the model was reduced in order, the LOOCV MSE decreased and reached a minimum with 22 input variables (Fig. 5A–C, Table 2). The 22-input variable PLS model provided the best predictive accuracy of any PLS model. The eliminated inputs are also considered to be factors that decrease or do not affect the predictive accuracy of the model. As the input variables were sequentially eliminated, the error increased and reached a level similar to that of the full PLS model when 5 input variables remained. The last 5-input variable model was denoted as the simple PLS model (Fig. 5D). The input variables in the simple PLS model were pERK at 10 min (pERK10), pCREB at 5 and 60 min (pCREB5 and pCREB60), pAKT at 5 min (pAKT5) and pJNK at 30 min (pJNK30). This result indicates that pERK10, pCREB5, pCREB60, pAKT5 and pJNK30 were the minimum set of the inputs that showed a comparable predictive accuracy as that of the entire data set of the outputs in the full PLS model with 60 input variables. We further validated the simple model by use of the inhibitor experiments in Figure 4 and found that the simple model similarly predicted the inhibitor experimental result as the full and best models (Fig. 6 and Fig. S1). The variables pERK10, pAKT5 and pJNK30 were considered to indicate the peak activity of pERK, pAKT and pJNK, respectively (Fig. 1). The variables pCREB5 and pCREB60 may correspond to pERK and PKA activity, respectively, because pCREB is regulated by pERK and PKA. We then plotted the loadings and scores of the 5 variables in the first and second principal component axes (Fig. 5E, F). To facilitate simple interpretation of the simple model with 5 input variables, we selected the single output variable for each output with maximum norm that is considered to indicate the maximum amount of information regarding the output variable. The selected output variables were cell death at 12 hour (Cell death12), cell cycle at 32 hour (Cell cycle32), JUNB at 270 min (JUNB270), FOSB at 180 min (FOSB180), c-FOS at 60 min (c-FOS60), neurite length at 48 hour (Neurite length48), cell viability at 48 hour (Cell viability48), c-JUN at 90 min (c-JUN90) and EGR1 at 90 min (EGR1-90). We plotted the loadings and scores of the selected single outputs for each variable (Fig. 5G, H) of the simple PLS model with 5 input and 9 output variables (Fig. 7A). The variable pJNK30 correlated with Cell death12 and pCREB5, and pCREB60 correlated with JUNB270, FOSB180 and c-FOS60. pERK10 and pAKT5 correlated with c-JUN90 and EGR1-90. Neurite length48 and Cell viability48 correlated with pCREB5 and pCREB60, and pERK10. Anisomycin, PACAP, NGF and EGF were plotted in the first, second, third and fourth quadrants, respectively. The results of the simple PLS model are consistent with those of the full PLS model, indicating that the simple PLS model effectively represents the relationships between the inputs and the outputs underlying cell-fate decisions in PC12 cells.

**Table 2:** The 22 input variables in the best model and five input variables in the simple model.

	2	5	10	15	30	60	90	120	180	270	360 (min)
pERK			B, S		B	B	B	B	B		
pCREB	B	B, S				B,S					B
pJNK	B	B		B	B,S	B			B		
pAKT		B, S			B	B					
pp38	B	B			B					B	

(B; variables in the Best PLS model; S; variables in the Simple PLS model).

## Discussion

In this study, we employed PLS regression to describe the relationship between the phosphorylation of signaling molecules and the expression of IEGs and cellular phenotypes, which has been applied to similar biological data sets [1]–[3], [26]–[28], [33]. The loadings and scores of the PLS model highlight characteristics of the MIMO system and growth factor specific input-output relationships of cell-fate decisions, respectively, in PC12 cells.

One of the technical highlights of this study is the model reduction via backward elimination PLS regression. We found that a reduction of the number of input variables provided better predictive ability, and the 22-variable model showed the best predictive ability. We further reduced the number of variables, and found that the simple PLS model with 5 input variables showed a comparable predictive ability to that of the full PLS model with 60 input variables. We also reduced the number of outputs by selecting the output with the maximum norm, which is considered to encode the maximum information of each output. Such reduction methods help provide an intuitive understanding of the complex relationships in the MIMO system and can be widely applied to any signaling and cellular phenotypes. The variable importance in the projection (VIP) scores is indicative of the importance of each variable in the projection used in a PLS model and are often used for variable selection [1]. We calculated the VIP scores of all variables in the full PLS model and found that the 5 variables in the simple PLS model were included in the top 20 variables selected by VIP (Table S4). Furthermore, we compared the simple PLS model with the VIPs-PLS model with 5 input variables selected from the highest VIP score (Table S5). The simple PLS model with 5 input variables showed lower LOOCV MSE and higher correlation than the VIPs-PLS models with 5 input variables. indicating that the prediction ability of the simple model is higher than the VIPs-PLS model. These results support the importance of these 5 variables in the simple PLS model.

The simple PLS model with 5 variables demonstrated a comparable predictive ability to that of the full PLS model. The simple PLS model with 5 input variables and 9 output variables (Fig. 7A) facilitates an intuitive understanding of the MIMO system and growth factor specific input-output relationships of cell-fate decision in PC12 cells (Fig. 7B). The simple model showed the similar predictive ability to the full model against the inhibitor experiment (Fig. 6 and Fig. S1), indicating the simple model essentially captures the input-output relationship of the MIMO system. The selected input variables in the simple PLS model were pERK10, pCREB5, pCREB60, pAKT5 and pJNK30. Among the selected inputs, pERK10, pAKT5 and pJNK30 correspond to their peak activities and are considered to encode the maximum information of the signaling molecules (Fig. 1). It has been reported that sustained ERK activity is required for cell differentiation in PC12 cells [9], [34], [35]; however, late pERK was not selected in the simple PLS model, although the model can capture the characteristics of the neurite length. This omission may occur because late pERK information is encoded by pCREB60 levels, a downstream molecule of ERK, in the simple PLS model. Two different time points of pCREB, pCREB5 and pCREB60, were selected, suggesting that pCREB5 and pCREB60 encode different information. The phosphorylation of CREB has been reported to be regulated by ERK and PKA [36]. NGF and EGF have been reported to induce the phosphorylation of CREB via ERK, and PACAP has been reported to induce the phosphorylation of PKA [10], [36], [37]. Therefore, pCREB5 and pCREB60 likely encode different information for stimuli and upstream molecules.

We found that the simple model shows the similar predictive ability for the inhibitor experiments to the full and best models, meaning that the selected 5 input variables in the simple model have high explanatory abilities to the outputs. We further examined the contribution of the 5 input variables in the simple model to the specific outputs. We made the best models for each output and compared to the selected input variables to those in the simple models (Table S7). The selected input variables in the best models for each output included different sets of the input variables in the simple model, suggesting that the different sets of the input variables in the simple model specifically contributed to each output. For example, the cell cycle and cell death shared the same set of the input variables such as pERK10, pJNK30 and pAKT5, suggesting that these output may share the same upstream dependency. Each IEG had the different sets of the selected input variables except c-FOS and FOSB, suggesting that the regulation of c-FOS and FOSB were similar, and the regulation of other IEGs were different. Moreover, pERK10, which was selected in the simple and best models, was also selected in the best models for each output except neurite length and c-JUN, suggesting that pERK10 is involved in multiple outputs. pERK30 to pERK180, which was selected in the best model, were also selected in the best models for the neurite length, cell cycle, c-FOS, c-JUN and EGR1. This result is consistent with the previous observations that sustained ERK activation regulates the cellular phenotypes [38] and protein expression of c-FOS, c-JUN and EGR1 [39]. These input variables were not selected in the simple model, possibly because pCREB60 reflect the sustained ERK activation in the simple model. pCREB5, which was selected in the simple and best models, was also selected in the best models for neurite length, cell death and JUNB, suggesting that pERK10 is involved in these outputs. pCREB60, which was selected in the simple and best models, was also selected in the best models for neurite length and cell cycle. pCREB60 in the simple model may reflect sustained ERK activation. pJNK30, which was selected in the simple and best models, was also selected in the best models for cell cycle, cell death, EGR1 and FOSB. The involvement of JNK in the regulation of cell cycle [40] and cell death [24] has been reported. The involvement of JNK in the regulation of EGR1 and FOSB are novel, and should be tested by experiment. pAKT5, which was selected in the simple and best models, was also selected in the best models for neurite length, cell cycle, cell death, c-FOS and FOSB. The negative relationship of pAKT5 to c-FOS and neurite length is novel and should be tested by experiment (Figure 7). We further performed the clustering analysis of the data in Table S7. The hierarchy levels of c-JUN, c-FOS and EGR1 are higher than those of cellular phenotype and JUNB and FOSB. Moreover, as the hierarchy level descends, the input variables of the lower hierarchy levels seem subsets of those of the higher hierarchy level. This result suggests that c-JUN, c-FOS and EGR1 are upstream regulators of cellular phenotypes and JUNB and FOSB, which is consistent of previous observations [41], [42]. Given that c-JUN, c-FOS and EGR1 are at the higher hierarchy levels and share the common input variables with cellular phenotype and JUNB and FOSB of the lower hierarchy level, the whole system is likely to be a MIMO system.

PLS regression reduces dimensionality of the inputs and outputs into respective latent variables. In this study, we further reduced the full PLS model by eliminating inputs variables from the original PLS model using the backward elimination method, and found that accuracy of the eliminated PLS model (best PLS model) was improved. These results demonstrate that the backward elimination PLS regression has two-fold advantage compared to the conventional PLS regression; reduction of input variables and improvement of accuracy of prediction. We eliminated the input variables and obtained the best model with input of 22 input variables, which shows the best accuracy of prediction. We further eliminated input variables and obtained the simple model with 5 input variables, which shows comparative predictive ability to the full model and facilitate interpretation identified 5 inputs as the minimum set of the inputs that characterized the MIMO system in PC12 cells. Thus, our data-driven statistical modeling method is widely useful to effectively extract simple relationships of the cellular MIMO system from large-scale data sets.

## Materials and Methods

### Antibodies

Mouse anti-phospho-ERK1/2 (Thr 202/Tyr 204) monoclonal antibody (mAb) (#9106), rabbit anti-phospho-CREB (Thr 133) mAb (#9198), rabbit anti-phospho-JNK (Thr183/Tyr185) mAb (#4668), rabbit anti-EGR1 mAb (#4154), rabbit anti-c-JUN mAb (#9165), rabbit anti-c-FOS mAb (#2250), rabbit anti-JUNB mAb (#3753), rabbit anti-FOSB mAb (#2251), and rabbit anti-cleaved Caspase 3 mAb (#9664) were purchased from Cell Signaling Technology (Beverly, MA). Rabbit anti-phospho p38 mAb (#v1211) was purchased from Promega (Madison, WI).

### Cell Culture and Treatments

PC12 cellswere cultured at 37°C under 5% CO_2_ in Dulbecco’s modified Eagle’s medium (DMEM) supplemented with 10% fetal bovine serum and 5% horse serum (Invitrogen, Carlsbad, CA) [43]. Cells were stimulated using recombinant mouse β-NGF (R&D Systems, Minneapolis, MN), EGF (Roche, Mannheim, Germany), PACAP (Sigma, Zwijndrecht, The Netherlands), or anisomycin (EMD Biosciences, Inc., San Diego, CA) as previously described [43]. We used a low dose of anisomycin (50 nM) to activate p38 and JNK without inhibiting translation. For the QIC assays, cells were seeded at a density of 10^4^ cells per well in 96-well poly-L-lysine–coated glass-bottomed plates (Thermo Fisher Scientific, Pittsburgh, PA) and then starved in DMEM containing 25 mM HEPES and 0.1% bovine serum albumin for approximately 18 h before stimulation. Cells seeded in 96-well microplates were stimulated by replacing the starvation medium with the medium containing the stimulant using a liquid handling system (Biomek® NX Span-8, Beckman Coulter, Fullerton, CA) with an integrated heater-shaker (Variomag®, Daytona Beach, FL) and robotic incubator (STX-40, Liconic, Mauren, Liechtenstein). All of the cells within a plate were fixed simultaneously to prevent their exposure to formaldehyde vapor during the treatment.

### QIC (Quantitative Image Cytometry)

QIC was performed as previously described [44]. Briefly, after growth factor stimulation, the cells were fixed, washed with phosphate-buffered saline (PBS), and permeabilized with blocking buffer (0.1% Triton X-100, 10% fetal bovine serum in PBS). The cells were then washed and incubated for 2 h with primary antibodies diluted in Can Get Signal immunostain Solution A (Toyobo, Osaka, Japan). The cells were washed three times and then incubated for 1 h with secondary antibodies. After immunostaining, the cells were treated for nucleus and cytoplasm staining by incubating with Hoechst 33342 (Invitrogen, Carlsbad, CA) and CellMask Deep Red stain (Invitrogen Carlsbad, CA), respectively. The images of the stained cells were acquired using a CellWoRx (Thermo Fisher Scientific, Pittsburgh, PA) automated microscope with a ×10 objective. For QIC analyses, we acquired two different fields for each well and obtained 1238±356 (mean ± SD) cells for each well. All liquid handling for the 96-well microplates was performed using a Biomek® NX Span-8 liquid handling system (Beckman Coulter, Fullerton, CA).

### Quantitative Analysis of the Neurite Length

PC12 cells (0.5×10^4^ cells/well) were fixed using a 10% formalin solution (Wako, Osaka, Japan) for 10 minutes. Cells were washed with phosphate-buffered saline (PBS), incubated with 1 µg/ml Hoechst 33342 solution (Life Technologies, Carlsbad, CA) and 1 µg/ml CellMask (Life Technologies) in PBS for 1 hour at room temperature and then washed with PBS. Images were captured using a CellWoRx microscope (Thermo Fisher Scientific, Rockford, IL). Using the CellMask signal as the neuronal cell image and the Hoechst signal as the nuclear image, the lengths of the neurites were measured with the NeuroTracer NIH ImageJ plug-in [45]. The length of the neurites of cells under each stimulation condition was represented as the averaged neurite lengths of cells.

### Cell Viability Assay (Mitochondrial Respiratory Chain Activity)

Cell viability was determined by measuring mitochondrial reduction of the MTS dye [3-(4,5-dimethythiazol-2-yl)-5-(3-carboxymethoxyphenyl)-2-(4-sulfophenyl)-2H-tetrazolium] reagent into a soluble formazan product (Promega, Madison, WI) for the quantification of the respiratory chain activity of the mitochondria. PC12 cells were plated on Poly-L-lysine (PLL)-coated 96-well plates. After incubation, cells were treated with MTS solution (1 mg/ml), and the intracellular soluble formazan produced by the cellular reduction of the MTS was determined by recording the absorbance of each 96-well plate using a Mithras LB940 microplate reader (Berthold Japan, Tokyo, Japan) at a wavelength of 490 nm.

### Cell Death Assay (Activity of Caspase 3)

Cell death was determined by measuring of activation of Caspase 3 as cleaved Caspase 3 using western blot assays. Cell lysates were subjected to standard sodium dodecyl sulfate polyacrylamide gel electrophoresis (SDS-PAGE). After fractionation by SDS-PAGE and transfer to nitrocellulose membranes, the blots were incubated with antibodies directed at Cleaved Caspase 3 (1∶1000 dilution; Cell Signaling Technology, Danvers, MA, #9664 ) or pan ERK1/2 (1∶2000 dilution; Cell Signaling Technology, #9102) followed by incubation with horseradish peroxidase-conjugated rabbit IgG (GE Healthcare, Buckinghamshire, England). Chemiluminescence was detected using Immobilon Western (Millipore, Billerica, MA). The resulting image was captured with a luminescent image analyzer LAS-4000 (Fujifilm, Tokyo, Japan). The signal intensity was quantified using Phoretix 1D software (TotalLab Ltd, Newcastle upon Tyne, UK).

### Cell Cycle Assay (Cell Cycle S-phase Fraction)

Cell cycle S-phase fraction was determined by the incorporation of 5-*ethynyl*-2′-deoxyuridine (EdU) using the Click-iT EdU Cell Proliferation AssayKit (Invitrogen). The PC12 cells were incubated with 10 µM EdU for 1 hour before fixation, permeabilization, and EdU staining, which were performed according to the kit manufacturer’s protocol. The proportion of nucleated cells incorporating EdU was determined by fluorescence microscopy using a CellWoRx microscope (Thermo Fisher Scientific), and the fraction of cells in the S-phase was measured using MATLAB software (MathWorks).

### Partial Least Squares Regression Method

The partial least squares regression method used in this study was described in a previous publication [1], [46]. Partial least squares regression is a predictive two-block regression method based on estimated latent variables and is applied for the simultaneous analysis of two data sets. The purpose of PLS regression is to build a linear model that enables the prediction of outputs from inputs. In this study, PLS regression analysis was performed using the MATLAB (Mathworks) software suite. Data were normalized by mean centering and variance scaling the different measurements.

Let 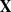 be the (20×60) inputs matrix for PLS modeling. The 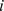-th (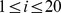) row vector of 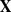 is the input vector 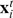 where 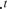 denotes the transpose of a vector or matrix . The input vector 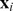 consists of 60 metric variables which are time course points of MAPKs and CREB. We used 20 doses of stimuli to obtain 20 samples as input vectors, hence, 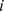 corresponds to the attribute of stimulation. Let 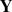 be the (20
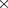
95) outputs matrix. The 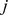-th (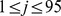) column vector 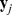 of 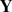 is the output vector of which each variable correspond to the attribute of stimulation, and 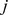 corresponds to the attribute of time course point of the IEGs or phenotype.

The PLS model can be understood as two steps regression model developed simultaneously. The first step can be considered as consisting of the development of outer relations (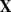 and 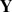 metric individually). These data matrix were decomposed in latent variables plus a residue matrix. The sub-matrices can be represented as the product of the scores and the loadings which can be re-grouped in independent matrices for the 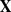 and 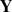 matrix as follows:
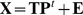


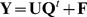



where 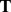 and 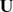 are the scores, and 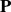 and 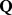 are the loadings, for the 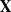 and 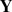 matrix, respectively. The matrices 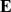 and 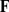 correspond to the residues associated with the PLS modeling. The second step is a linear inner relation linking between 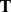 and 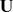,
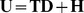
where 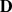 is the diagonal matrix and 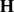 denotes the residual matrix.

Eventually, PLS regression is yielded by.
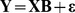
where 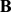 is the matrix of regression coefficients



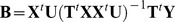
and 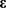 is the residual matrix.

The optimum number of components were determined by minimizing MSE of Leave-one-out cross-validation as LOOCV MSE [30],

LOOCV MSE = 
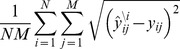


where 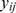 is the 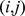 variables of the (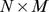) matrix 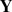 and 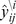 is the prediction for 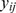 by PLS model which was trained by the data set removed 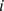-th sample.

### Backward Elimination PLS Regression

We applied a backward elimination variable selection method [32] for PLS because backward elimination can improve the accuracy of a PLS model. The backward elimination PLS regression began with the full PLS model with the input vector of *M* variables. We define the LOOCV MSE removing 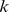-th variable from input vector as
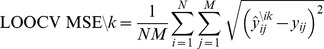
where 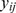 is the 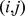 element of the (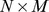) matrix 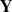 and 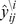 is the prediction for 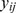 by PLS model which was trained by the data set removed 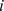-th sample. In the 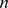-th step of procedure, the elimination 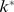-th variable of input vector was determined by minimizing LOOCV MSE 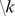 for 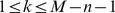




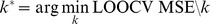
then, 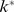-th variable was eliminated from the input vector, and redefined the new input vector of which 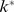-th variable was eliminated for 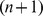-th step. We iterated this procedure until the 4 variables remained, which is the same number as the principal components.

### Variable Importance in Projection (VIP)

We calculated the *Variable Importance in Projection* (VIP) of [47] to summarize each variable contribution to the model. VIP describes which ***X*** variables characterize the ***X*** block well and which variables correlate with ***Y***. VIP values summarize the overall contribution of each input variable to the PLS model, summed over all components and weight according to the ***Y*** variation accounted for by each component. VIP is calculated as follows:
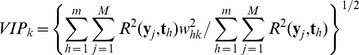
for each *k*-th input variable *k = 1,*…, *p,* where 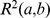 stands for the squared correlation between items in vector ***a*** and ***b***, 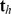 is the *h-*th column vector of the score matrix ***T***, *m* is the number of principle components, and 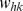 is the (*h,k*) element of the weight matrix ***W***.

## Acknowledgments

We thank Kevin Janes (University of Virginia) for technical advice with the PLS regression and helpful discussions. We also thank our laboratory members, Kanako Watanabe, Takeshi Saito, Takamasa Kudo and Risa Kunihiro, for critically reading this manuscript and their technical assistance with the experiments.

## Supporting Information

Figure S1 **The correlation coefficient of the full, best and simple PLS model between the measured outputs and predicted outputs using the inhibitor experiments.**
(TIF)

Figure S2 **Time courses of the signaling molecules, IEGs and cellular phenotypes in response to NGF (0.15 ng/ml), PACAP (1 nM) and simultaneous addition of NGF (0.15 ng/ml) and PACAP (1 nM).**
(TIF)

Table S1 **All data sets used for construction of the full PLS model.**
(XLS)

Table S2 **Experimental data for the inhibitor experiments used for the PLS model validation.**
(XLS)

Table S3 **Coefficient matrices of the full PLS model, the best PLS model and the simple PLS model.**
(XLS)

Table S4 **The VIP scores of the input variables.**
(EPS)

Table S5 **The correlation coefficients and LOOCV MSE in the backward elimination PLS models and the VIPs-PLS model with 5 input variables.**
(EPS)

Table S6 **The correlation cofficients between the full PLS model and the experiments with simultaneous addition of NGF and PACAP.**
(EPS)

Table S7 **The best models for each output.**
(EPS)
