# Supplementary figures and images for: The Extraction of Simple Relationships in Growth Factor-Specific Multiple-Input and Multiple-Output Systems in Cell-Fate Decisions by Backward Elimination PLS Regression
Source: PLoS One. 2013 Sep 9;8(9):e72780. doi: 10.1371/journal.pone.0072780 (PMC3767677; doi:10.1371/journal.pone.0072780)

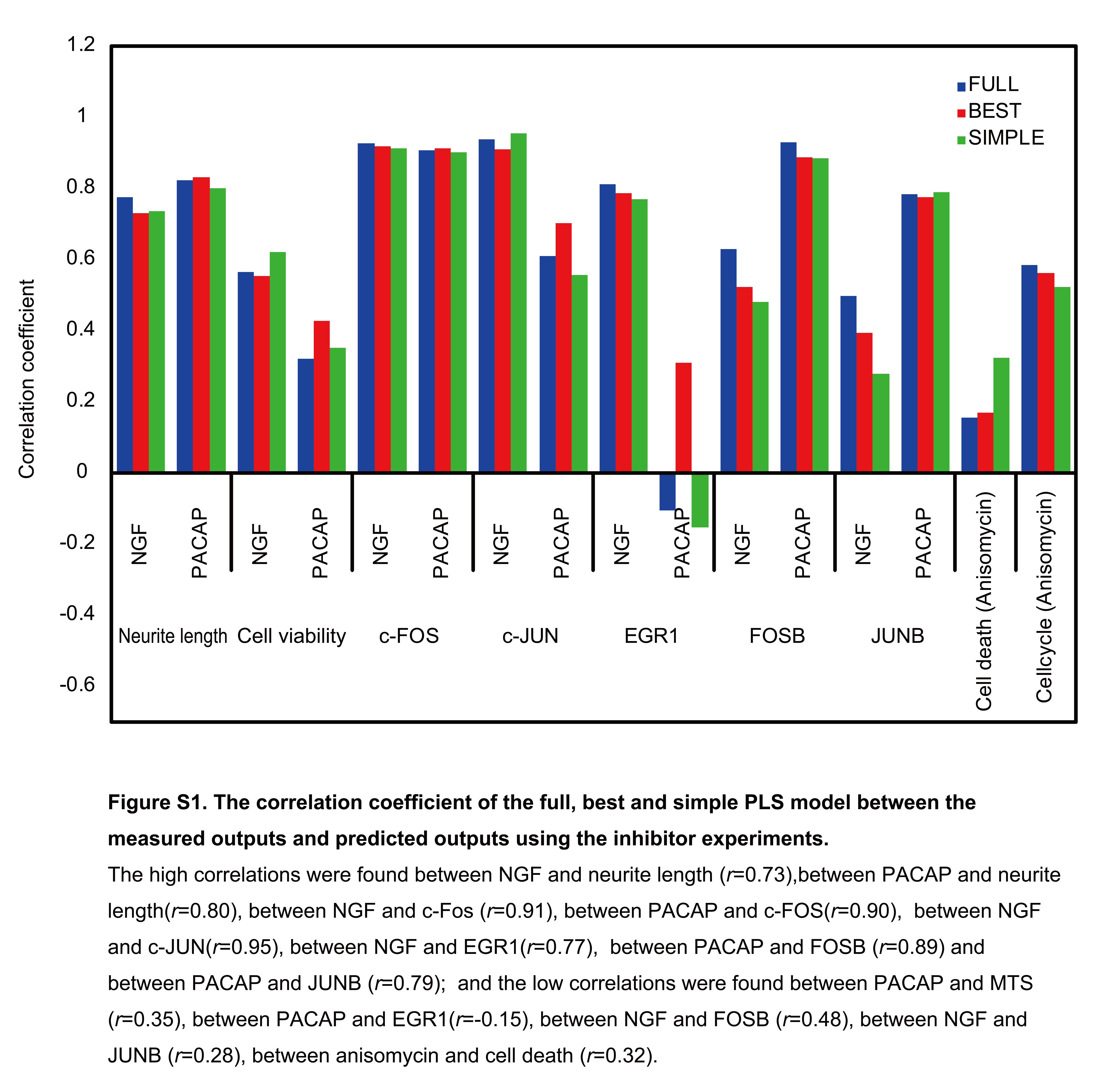

Supplement: Figure S1 — The correlation coefficient of the full, best and simple PLS model between the measured outputs and predicted outputs using the inhibitor experiments. (TIF) [file pone.0072780.s001.tif]
